# Supplementary material for: Model-based fMRI reveals dissimilarity processes underlying base rate neglect
Source: eLife. 2018 Aug 3;7:e36395. doi: 10.7554/eLife.36395 (PMC6108825; doi:10.7554/eLife.36395)
Supplement: Supplementary file 1. [file elife-36395-supp1.docx]

| Subject # | Common Responses | Rare Responses |
| --- | --- | --- |
| *1* | 12 | 6 |
| *2* | 3 | 21 |
| *3* | 13 | 11 |
| *4* | 12 | 10 |
| *5* | 12 | 10 |
| *6* | 16 | 7 |
| *7* | 15 | 9 |
| *8* | 10 | 12 |
| *9* | 2 | 22 |
| *10* | 6 | 14 |
| *11* | 13 | 10 |
| *12* | 14 | 10 |
| *13* | 11 | 13 |
| *14* | 10 | 14 |
| *15* | 10 | 13 |
| *16* | 15 | 8 |
| *17* | 7 | 17 |
| *18* | 7 | 10 |
| *19* | 20 | 3 |
| *20* | 11 | 13 |
| *21* | 15 | 7 |
| *22* | 10 | 14 |
